# Artificial de novo biosynthesis of hydroxystyrene derivatives in a tyrosine overproducing *Escherichia coli* strain

**DOI:** 10.1186/s12934-015-0268-7

**Published:** 2015-06-10

**Authors:** Sun-Young Kang, Oksik Choi, Jae Kyoung Lee, Jung-Oh Ahn, Jong Seog Ahn, Bang Yeon Hwang, Young-Soo Hong

**Affiliations:** Chemical Biology Research Center, Korea Research Institute of Bioscience and Biotechnology, 30 Yeongudanji-ro, Ochang-eup, Chungbuk, 363-883 Republic of Korea; Biotechnology Process Engineering Center, Korea Research Institute of Bioscience and Biotechnology, 30 Yeongudanji-ro, Ochang-eup, Chungbuk, 363-883 Republic of Korea; Department of Pharmacy Graduate School, Chungbuk National University, Cheongju, 361-763 Republic of Korea

**Keywords:** 4-Hydroxystyrene, 3,4-Dihydroxystyrene, 4-Hydroxy-3-methoxystyrene, de novo Biosynthesis

## Abstract

**Background:**

Styrene and its derivatives as monomers and petroleum-based feedstocks are valuable as raw materials in industrial processes. The chemical reaction for styrene production uses harsh reaction conditions such as high temperatures or pressures, or requires base catalysis with microwave heating. On the other hand, production of styrene and its derivatives in *Escherichia coli* is an environmental friendly process to produce conventional petroleum-based feedstocks.

**Results:**

An artificial biosynthetic pathway was developed in *E. coli* that yields 4-hydroxystyrene, 3,4-dihydroxystyrene and 4-hydroxy-3-methoxystyrene from simple carbon sources. This artificial biosynthetic pathway has a codon-optimized phenolic acid decarboxylase (*pad*) gene from *Bacillus* and some of the phenolic acid biosynthetic genes. *E. coli* strains with the *tal* and *pad* genes, the *tal*, *sam5*, and *pad* genes, and the *tal*, *sam5*, *com*, and *pad* genes produced 4-hydroxystyrene, 3,4-dihydroxystyrene and 4-hydorxy-3-methoxystyrene, respectively. Furthermore, these pathways were expressed in a tyrosine overproducing *E. coli*. The yields for 4-hydroxystyrene, 3,4-dihydroxystyrene and 4-hydorxy-3-methoxystyrene reached 355, 63, and 64 mg/L, respectively, in shaking flasks after 36 h of cultivation.

**Conclusions:**

Our system is the first to use *E. coli* with artificial biosynthetic pathways for the de novo synthesis of 3,4-dihydroxystyrene and 4-hydroxy-3-methoxystyrene in a simple glucose medium. Similar approaches using microbial synthesis from simple sugar could be useful in the synthesis of plant-based aromatic chemicals.

**Electronic supplementary material:**

The online version of this article (doi:10.1186/s12934-015-0268-7) contains supplementary material, which is available to authorized users.

## Background

Styrene is one of the most important aromatic chemicals produced industrially. It has many uses including in the manufacture of polystyrenes, plastics, and styrene-butadiene rubbers. Hydroxystyrene is also a monomer used in the production of numerous polymers and in petroleum-based feedstocks for resins, elastomers, and adhesives. Poly-hydroxystyrene, also called polyvinylphenol (PVP), is a plastic structurally similar to polystyrene. PVP is used in electronics as a dielectric layer in organic transistors of organic thin-film-transistor liquid–crystal display. Its ability to form linear polymers and its excellent solubility in organic solvents make hydroxystyrene a good reagent in the chemical synthesis of various coatings for electronic devices, such as a photoresist [1]. Currently, styrene production predominantly comes from the energy-intensive chemocatalytic dehydrogenation of petroleum-derived ethylbenzene [2, 3]. Because of concerns over depleting feedstock availability and deleterious environmental impacts, a bio-based method could be a low energy, renewable alternative to petroleum-derived styrene [4]. Thus, an artificial pathway for styrene and hydroxystyrene biosynthesis from glucose in *Escherichia coli* was previously engineered [5–7]. 4-Hydroxystyrene is also produced in the solvent-tolerant *Pseudomonas putida* strains, originally designed for phenol and 4-coumarate production [8]. In addition, styrene production in *Saccharomyces cerevisiae* was recently reported combining metabolic evolution with systematic strain and pathway engineering [9, 10].

In recent years, several of artificial biosynthetic pathways have been engineered in microorganisms to produce useful, functionalized phenolic compounds from glucose [4, 6, 11–15]. In our laboratory, we investigate artificial biosynthetic pathways in microorganisms to produce a number of useful phenylpropanoids from plants [16–18]. Many of these substances in the phenylpropanoid pathway is phenolic acids, e.g., cinnamic, 4-coumaric, caffeic, ferulic, and sinapic acids. Their abundance has garnered much interest in their use to produced novel flavors, fragrances, pharmaceuticals and other chemicals [19].

**Figure 1:**
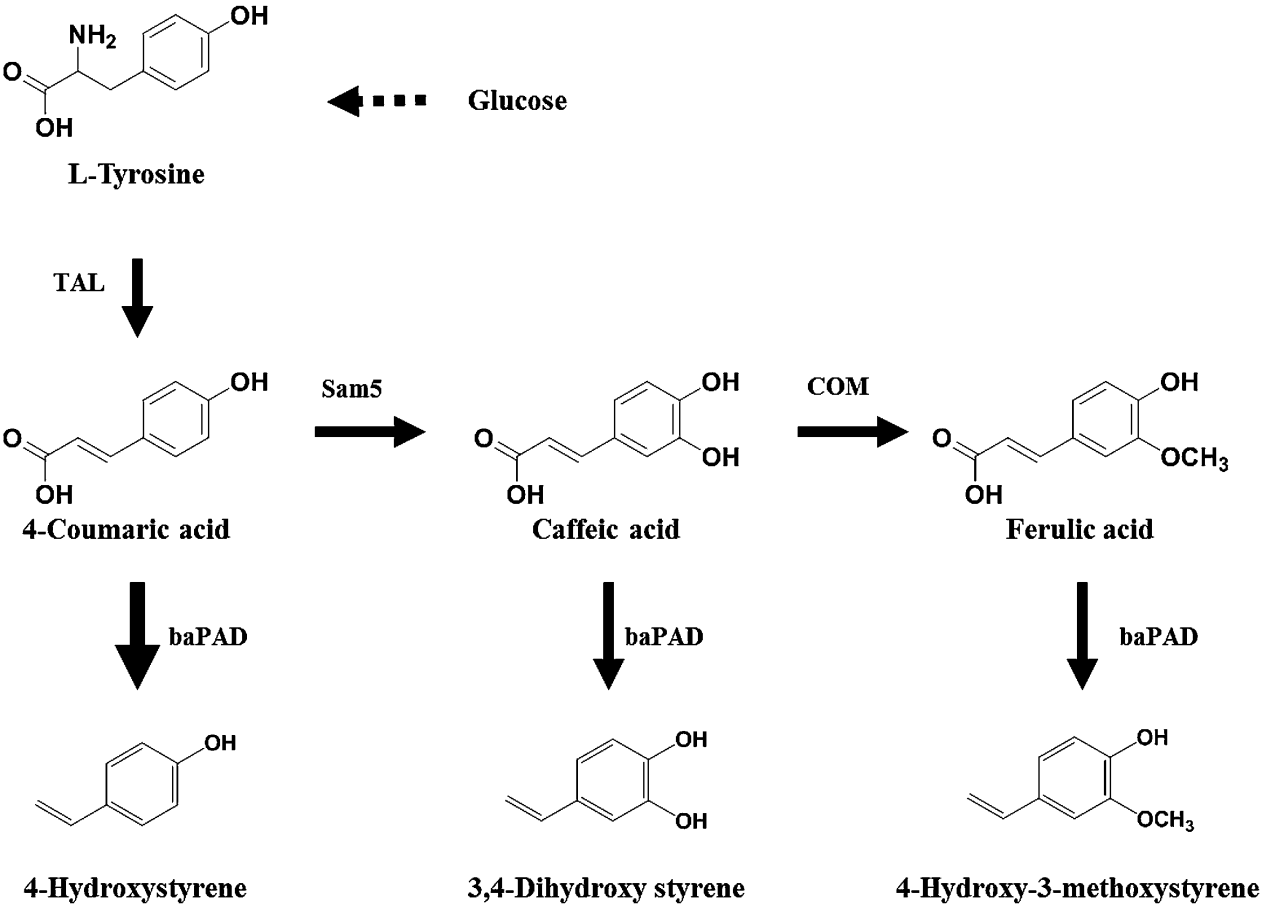
Engineered biosynthetic pathways for hydroxystyrene derivatives starting from tyrosine in *E. coli.*

The proposed hydroxystyrene biosynthesis pathway uses endogenously synthesized l-tyrosine as a precursor which is converted to hydroxystyrenes through a series of enzymatic steps shown in Figure 1. Phenolic acids, as key intermediates, have been previously reported for a series of phenolic acid biosynthetic genes characterized in *E. coli* [20]. The next step in the proposed hydroxystyrene biosynthesis pathway involves the subsequent decarboxylation of phenolic acids by a phenolic acid decarboxylase, which converts these acids to their styrene derivatives [21–23].

This study constructed an artificial biosynthesis pathway to produce hydroxystyrene with the tyrosine ammonia lyase gene (*tal*) from *Saccharothrix espanaensis* and phenolic acid decarboxylase gene (*pad*) from *Bacillus amyloliquefaciens*. Additionally, serial artificial biosynthetic gene expression sets were developed and used to produce 3,4-dihydroxystyrene and 4-hydorxy-3-methoxystyrene by adding 4-coumarate 3-hydroxylase gene (*sam5*) and caffeic acid methyltransferase gene (*com*), respectively. Then, an *E. coli* strain capable of high-level tyrosine production was constructed with the feedback-inhibition 3-deoxy-d-arabinoheptulosonate-7-phosphate (DAHP) synthase gene (*aroG*) and the chorismate mutase/prephenate dehydrogenase gene (*tyrA*) by modifying a previously reported expression system. This strain was highly optimized to produce phenolic acids with a heavily increased metabolic flux toward l-tyrosine. Production of both 4-hydroxystyrene and 3,4-dihydroxystyrene from glucose was about 20-fold higher in the engineered tyrosine overproducing *E. coli* strain compared to that of the wild type *E. coli*. Finally, the yields for 4-hydroxystyrene, 3,4-dihydroxystyrene and 4-hydorxy-3-methoxystyrene were 355, 63, and 64 mg/L, respectively, in shaking flasks after 36 h of cultivation. This is the first report of a de novo biosynthesis yielding 3,4-dihydroxystyrene and 4-hydroxy-3-methoxystyrene using a single vector system combining phenolic acid biosynthetic genes and phenolic acid decarboxylase gene in *E. coli*. In this study, the production of 4-hydroxystyrene and its derivatives is accomplished with a carbohydrate feedstock through serial artificial biosynthetic pathways in *E. coli* strains.

## Results and discussion

### Bioconversion of phenolic acids to hydroxystyrenes through phenolic acid decarboxylase

**Figure 2:**
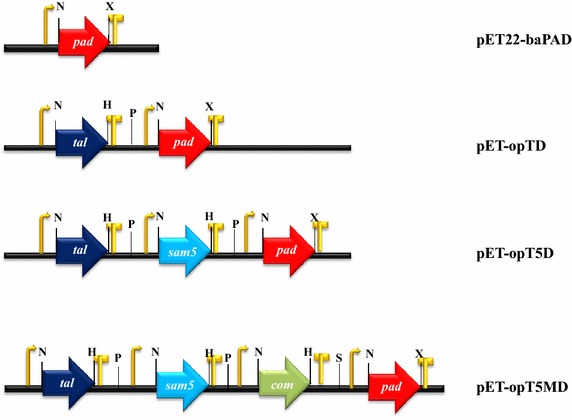
Expression vectors for the phenolic acid decarboxylase *pad* gene (pET22-baPAD) and organization of the artificial gene clusters used to produce each hydroxystyrene derivative in *E. coli*. All constructs contained the T7 promoter, RBS in front of each gene, and T7 terminator at the rear of each gene. P *Pac*I, S *Spe*I, N *Nde*I, H *Hind*III, X *Xho*I.

**Figure 3:**
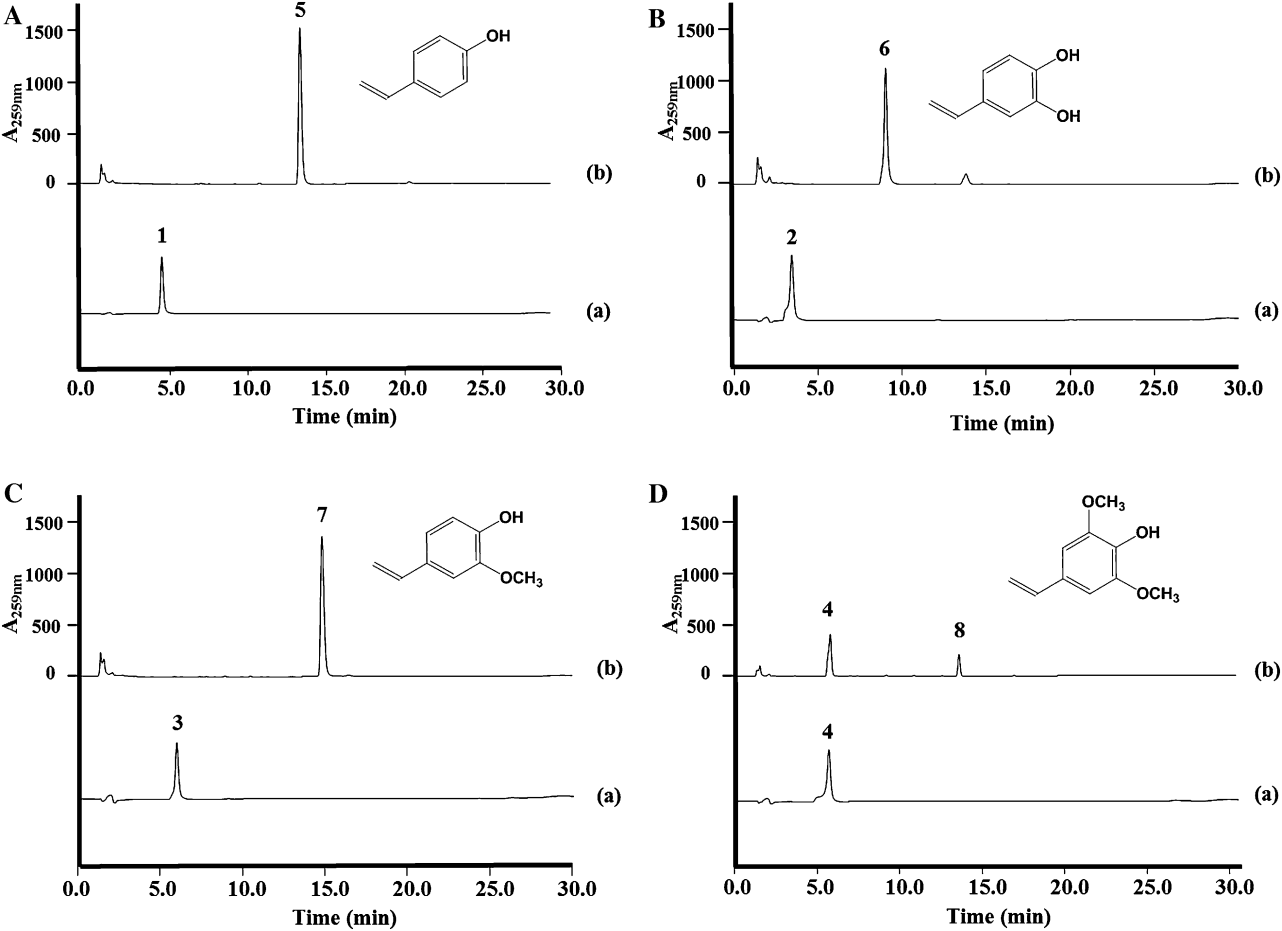
Bioconversion experiments with *E. coli* harboring pET22-baPAD fed with each phenolic acid. HPLC profiles of the 4-coumaric acid (**A**), caffeic acid (**B**), ferulic acid (**C**), and sinapic acid (**D**) supplemented *E. coli* harboring pET22-baPAD; *Peak 1* 4-coumaric acid, *peak 2* caffeic acid, *peak 3* ferulic acid, *peak 4* sinapic acid, *peak 5* 4-hydroxystyrene, *peak 6* 3,4-dihydroxystyrene, *peak 7* 4-hydroxy-3-methoxystyrene and *peak 8* 4-hydroxy-3,5-dimethoxystyrene.

Previous studies reported that phenolic acid decarboxylase from *B. amyloliquefaciens* produces 4-hydroxystyrene, 3,4-dihydroxystyrene and 4-hydroxy-3-methoxystyrene using 4-coumaric acid, caffeic acid and ferulic acid as substrates, respectively [21]. This study investigated the functions of the codon-optimized *pad* gene in *E. coli* in a bioconversion experiments using cinnamic acid, 4-coumaric acid, caffeic acid, ferulic acid, and sinapic acid as substrates. The five phenolic acids were added to cultures of *E. coli* C41(DE3) harboring the phenolic acid decarboxylase expression vector (pET22-baPAD) to investigate whether phenolic acid decarboxylase in vivo could produce styrene derivatives (Figure 2). The culture broths and bacterial cells were collected after 36 h of culturing, HPLC and GC/MS analyses were done using the samples. In the bioconversion conditions, almost of the 4-coumaric acid, caffeic acid and ferulic acid was consumed in the culture media, and each hydroxystyrene was detected as a main peak on the HPLC profiles (Figure 3). The detailed results of the GC/MS analyses are presented in the additional supporting section (Additional file 1: Figure S1). When the bioconversion rate was calculated through a quantitative comparison of the feeding substrates (2 mM) and the conversion ratios for 4-coumaric acid, caffeic acid, ferulic acid, and sinapic acid in *E. coli* with the *pad* gene were roughly 41, 27, 28, and 3%, respectively. In addition, the *pad* gene did not show any activity for cinnamic acid. These bioconversion ratios are consistent with the results of Jung et al. [21]. Interestingly, Jung et al. reported that the *pad* gene did not show any activity for sinapic acid. This study detected a 4-hydroxy-3,5-dimethoxystyrene peak on the HPLC profile (Figure 3D) and also confirmed its molecular weight with GC/MS analysis (Additional file 1: Figure S1). Our in vivo result for sinapic acid do not agree with the previously reported in vitro results. We do not know the reason for this, but it could be possible that the expressed PAD protein in *E. coli* affected the activity in vivo system.

### Construction of artificial biosynthetic pathways in *E. coli* to produce hydroxystyrene derivatives

To produce hydroxystyrene derivatives in *E. coli* with a simple sugar as a substrate, a series of plasmids were constructed containing artificial biosynthetic pathways that included the *pad* gene (Figure 2). We previously reported the production of 4-coumaric acid, caffeic acid, and ferulic acid in *E. coli* harboring artificial biosynthetic gene clusters in which the tyrosine ammonia lyase (*tal*) and 4-coumarate 3-hydroxylase (*sam5*) from *S. espanaensis* and caffeic acid methyltransferase (*com*) from *Arabidopsis thaliana* [20]. The artificial biosynthetic plasmids containing the additional *pad* gene for the hydroxystyrene derivatives were constructed as previously described methods [17]. In this study, the *pad* gene was under the control of the T7 promoter. A DNA fragment containing the promoter, the *pad* coding region, and the terminator was amplified using the pET22-baPAD plasmid as a template. The amplified *pad* fragment was ligated into the pET-opTAL, -opT5 and -opT5M plasmids containing the *tal* gene, the *tal* and *sam5* genes, and the *tal*, *sam5*, and *com* genes and designated as pET-opTD, -opT5D and -opT5MD plasmids, respectively (Figure 2). The genes each have their own T7 promoter, ribosome-binding site (RBS), and terminator sequence same as the parental vector.

**Figure 4:**
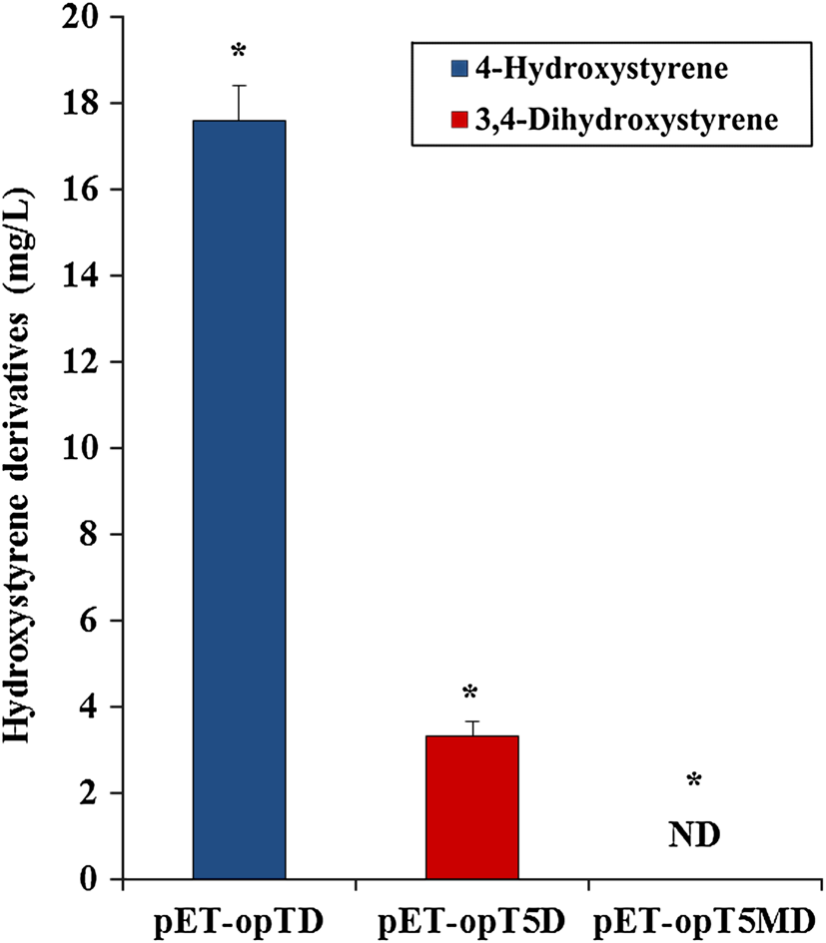
Production of hydroxystyrene derivatives by wild type *E. coli* strains with engineered expression vectors. The productivity of 4-hydroxystyrene (*blue bar*) and 3,4-dihydroxystyrene (*red bar*) with pET-opTD and pET-opT5D in the wild type *E. coli* C41(DE3) strain. *Error bars* indicate standard errors of the means (n = 3). The code ‘*ND*’ indicates that something was not detected in HPLC analysis. The production of 4-hydroxystyrene, 3,4-dihydroxystyrene, 4-hydroxy-3-methoxystyrene in wild type *E. coli* C41(DE3) were compared with single-factor ANOVA (P < 0.05) using Microsoft Excel. A *different letter code* (*asterisk*) indicates significant difference (P = 2.38E−08).

*E. coli* cells with the artificial biosynthetic pathways were cultured in a modified synthetic medium [24] to produce hydroxystyrene derivatives. The fermentation products of the *E. coli* strains with the *tal* and *pad* genes, the *tal*, *sam5* and *pad* genes and *tal, sam5*, *com,* and *pad* genes had new peaks on the HPLC profiles. The new peak at 15.0 min from the *E. coli* C41(DE3) strain with the *tal* and *pad* genes was identical to the standard for 4-hydroxystyrene, and the new peak at 9.5 min from the *E. coli* strain with the *tal*, *sam5*, and *pad* genes was identical to the standard for 3,4-dihydroxystyrene (Additional file 1: Figure S2). Contrary to our expectations, a peak for 4-hydroxy-3-methoxystyrene was not detected from the *E. coli* C41(DE3) strain with the *tal*, *sam5*, *com*, and *pad* genes after 36 h of culturing. Instead, an unknown peak appeared at 14 min on the HPLC profile, and the area of peak increased over time (Additional file 1: Figure S3). The UV spectrum of the unknown peak did not match any of the styrene derivatives including 4-hydroxystyrene and 4-hydroxy-3-methoxystyrene. However, a peak at 15.2 min was detected for 4-hydroxy-3-methoxystyrene after 2 h of culturing but disappeared after 12 h of culturing (Additional file 1: Figure S3). The *E. coli* C41(DE3) strain with the *tal* and *pad* genes (pET-opTD) yielded 17.6 ± 0.8 mg/L of 4-hydroxystyrene. The *E. coli* C41(DE3) strain with the *tal, sam5,* and *pad* genes (pET-opT5D) yielded 3.3 ± 0.3 mg/L of 3,4-dihydroxystyrene (Figure 4). However, the wild type *E. coli* strain with the *tal, sam5*, *com,* and *pad* genes (pET-opT5MD) produced only a trace amount of 4-hydroxy-3-methoxystyrene after 2 h of culturing, and the product was not detected after 36 h of culturing (Figure 4).

### Improved production of l-tyrosine-derived phenolic acids in a tyrosine overproducing *E. coli* strain

Because tyrosine serves as an immediate endogenous precursor to the hydroxystyrene pathway, its over production in *E. coli* is essential for hydroxystyrene biosynthesis. To develop a tyrosine overproducing *E. coli* strain, a classical metabolic engineering strategy was used. In *E. coli*, the aromatic amino acid biosynthesis pathway starts with the condensation of phosphoenolpyruvate and erythrose-4-phosphate, catalyzed by 3-deoxy-D-arabinoheptulosonate-7-phosphate (DAHP) synthase present in three isoforms with each feedback-regulated by aromatic amino acids. Subsequently, l-tyrosine biosynthesis from chorismate is catalyzed by the bifunctional enzyme chorismate mutase/prephenate dehydrogenase and the aromatic amino acid transaminase. Recently, l-tyrosine excreting *E. coli* strains were produced by deregulating the aromatic amino acid biosynthesis pathway [20]. A high-copy number vector with feedback-inhibition resistant (fbr) derivatives of DAHP synthase (*aroG*^*fbr*^) and chorismate mutase/prephenate dehydrogenase (*tyrA*^*fbr*^) genes [25] was overexpressed in an *E. coli* ΔtyrR strain [20]. In this study, the l-tyrosine producer, *E. coli* ΔCOS1, was engineered on the genome to also overexpress the *aroG*^*fbr*^ and *tyrA*^*fbr*^ genes in a ΔtyrR strain background. The *aroG*^*fbr*^ and *tyrA*^*fbr*^ gene cassette with a strong inducible T7 promotor was inserted into the *tyrR* gene region to make a stable strain for fermentation (Additional file 1: Figures S4, S5). Overall, greater improvements in l-tyrosine production were achieved initially in wild type *E. coli* (Additional file 1: Figure S6). A maximum yield of ~450 mg/L of l-tyrosine was produced by the *E. coli* ΔCOS1 strain in shaken flask experiments. The tyrosine yield was comparable to the tyrosine overproducing *E. coli* strain with the *aroG*^*fbr*^ and *tyrA*^*fbr*^ gene cassette vector (400 mg/L) as previously reported [20]. This ΔCOS1 strain was optimized for the production of aromatic compounds resulting in a heavily increased metabolic flux towards l-tyrosine. Therefore, it is suitable platform strain for the production of other l-tyrosine-derived phenolic acids including 4-coumaric acid, caffeic acid and ferulic acid, and hydroxystyrene derivatives using the phenolic acids as precursors.

**Figure 5:**
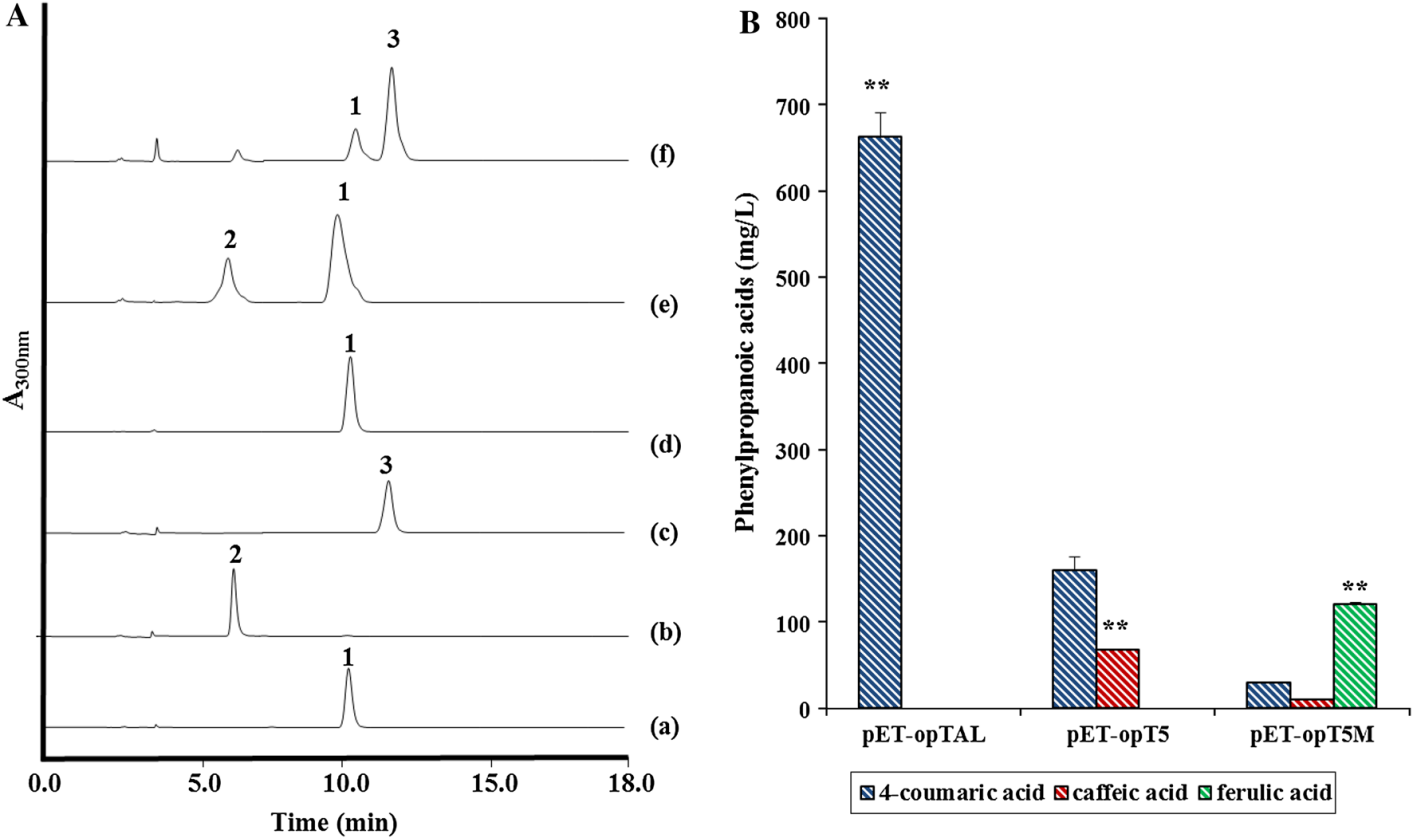
Production of phenolic acids by engineered tyrosine overproducing *E. coli* ΔCOS1 with engineered expression vectors. **A** HPLC profile of the standard 4-coumaric acid (*a*), caffeic acid (*b*), and ferulic acid (*c*); the culture broth of tyrosine overproducing *E. coli* ΔCOS1 harboring pET-opTAL (*d*); pET-opT5 (*e*); pET-opT5 M (*f*). The absorbance was monitored at 300 nm. *Peak 1* 4-coumaric acid, *peak 2* caffeic acid, *peak 3* ferulic acid. **B** The production of 4-coumaric acid (*diagonal blue*), caffeic acid (*diagonal red*), ferulic acid (*diagonal green*) with pET-opTAL, pET-opT5 and pET-opT5 M in mutant ΔCOS1 strain. *Error bars* indicate standard errors of the means (n = 3). The production of 4-coumaric acid, caffeic acid and ferulic acid in ΔCOS1 were compared with single-factor ANOVA (P < 0.05) using Microsoft Excel. A *different letter code* (*double asterisk*) indicates significant difference (P = 2.25E−08).

Using the same experimental conditions as above, the tyrosine-overproducing strains acquired a substantial capacity for 4-coumaric acid, caffeic acid and ferulic acid biosynthesis. As seen in Figure 4, the tyrosine overproducing *E. coli* ΔCOS1 strain expressing the *tal* gene (pET-opTAL) produced more than 662 ± 29 mg/L of 4-coumaric acid with an increase of 460% over the parental strain. The titers for caffeic acid did not improve significantly (67 ± 9 mg/L) compared to the titers for 4-coumaric acid, despite the 478% improvement over the parental strain [20]. On the other hand, ferulic acid synthesis in the ΔCOS1 strain expressing the *tal*, *sam5*, and *com* genes (pET-opT5 M) had a yield of 120 ± 3 mg/L. At the same time, an expected amount of accumulated 4-coumaric acid and caffeic acid was also identified. In agreement with previous results [20], the titers for ferulic acid were also increased over caffeic acid in the tyrosine-overproducing strain. It is possible that the metabolic pathway for ferulic acid could be alleviating any restrictions for the accumulation of caffeic acid in the cells. Finally, almost 700 mg/L of 4-coumaric acid was produced. Maximum titers of 76 and 123 mg/L for caffeic acid and ferulic acid were achieved, respectively (Figure 5). Thus, the results of this study suggest that in a tyrosine-overproducing strain system, higher titers of artificial phenolic acids from the biosynthetic pathways mentioned above guarantee higher concentrations of hydroxystyrene derivatives.

### Improved production of hydroxystyrene derivatives in tyrosine overproducing strains

**Figure 6:**
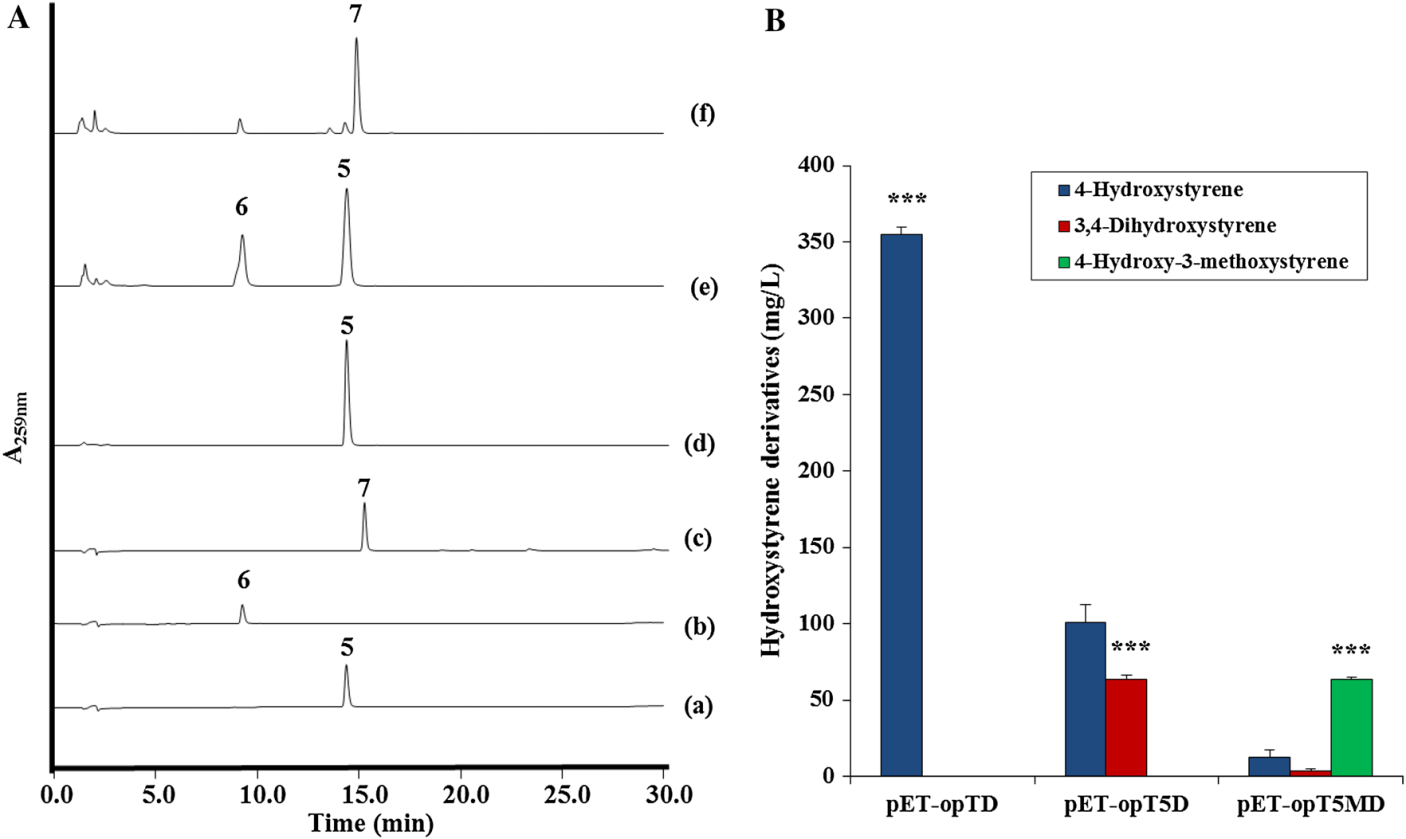
Production of hydroxystyrene derivatives by engineered tyrosine overproducing *E. coli* ΔCOS1 with engineered expression vectors. **A** HPLC profiles of standard 4-hydroxystyrene (*a*); 3,4-dihydroxystyrene (*b*); 4-hydroxy-3-methoxystyrene (*c*); the culture broth of tyrosine overproducing *E. coli* harboring pET-opTD (*d*); pET-opT5D (*e*); pET-opT5MD (*f*). The absorbance was monitored at 259 nm. *Peak 5* 4-hydroxystyrene, *peak 6* 3,4-dihydroxystyrene, *peak* *7* 4-hydroxy-3-methoxystyrene. **B** The productivity of 4-hydroxystyrene (*blue bar*), 3,4-dihydroxystyrene (*red bar*), and 4-hydroxy-3-methoxystyrene (*green bar*) with pET-opTD, pET-opT5D, and pET-opT5MD in ΔCOS1 strain. *Error bars* indicate standard errors of the means (n = 3). The production of 4-hydroxystyrene, 3,4-dihydroxystyrene, 4-hydroxy-3-methoxystyrene in ΔCOS1 were compared with single-factor ANOVA (P < 0.05) using Microsoft Excel. A *different letter code* (*triple asterisk*) indicates significant difference (P = 5.66E−11).

Next, the fermentation products of the *E. coli* C41(DE3) strain and the tyrosine-overproducing ΔCOS1 strain were compared each with the *tal* and *pad* (pET-opTD), *tal*, *sam5* and *pad* (pET-opT5D), and *tal*, *sam5*, *com*, and *pad* (pET-opT5MD) genes, respectively. With the same experimental conditions, the overproducing-tyrosine strains had a substantial synthesis capacity for 4-hydroxystyrene, 3,4-dihydroxystyrene and 4-hydroxy-3-methoxystyrene. As seen in Figure 6, the ΔCOS1 strain expressing the *tal* and *pad* genes (pET-opTD) produced 355 mg/L of 4-hydroxystyrene compared to the 17.6 mg/L produced in the C41(DE3) strain with the same genes. The ΔCOS1 strain expressing the *tal*, *sam5* and *pad* genes (pET-opT5D) produced 63 mg/L of 3,4-dihydroxystyrene compared to the 3.3 mg/L produced in the C41(DE3) strain with the same genes. Interestingly, 64 mg/L of 4-hydroxy-3-methoxystyrene was produced in only the ΔCOS1 strain expressing the *tal*, *sam5*, *com*, and *pad* genes (pET-opT5MD) with no yields in the C41(DE3) strain expressing the same genes after 36 h of culturing (Figure 4). An unknown peak at 14 min was also detected in addition to the 4-hydroxy-3-methoxystyrene peak from the ΔCOS1 strain. At the same time, a small amount (12 and 4 mg/L) of accumulated 4-hydroxystyrene and 3,4-dihydroxystyrene, respectively, was also identified in the ΔCOS1 strain expressing the *tal*, *sam5*, *com*, and *pad* genes (pET-opT5MD). To achieve higher yields of the hydroxystyrene derivatives, metabolite pattern analyses were performed for the tyrosine-overproducing ΔCOS1 strains expressing the *tal* and *pad* (pET-opTD), *tal*, *sam5* and *pad* (pET-opT5D), and *tal*, *sam5*, *com*, and *pad* genes (pET-opT5MD) according to culture times at 120, 40 and 50 h, respectively, until the production of each hydroxystyrene derivative was saturated. The amount of 4-hydroxystyrene was about 572 mg/L at 120 h while that for 3,4-dihydroxystyrene and 4-hydroxy-3-methoxystyrene were about 65 mg/L at 40 h and 64 mg/L at 36 h, respectively (Additional file 1: Figure S7). The production of 4-hydroxystyrene and 3,4-dihydroxystyrene increased by 20-fold and 21-fold compared to the parental strains, respectively. Furthermore, only the ΔCOS1 strain expressing the *tal*, *sam5*, *com*, and *pad* genes (pET-opT5MD) produced 64 mg/L of 4-hydroxy-3-methoxystyrene with no yields in the C41(DE3) strain expressing the same genes. When comparing the established baseline production of 4-hydroxystyrene from a previously reported *E. coli* platform that used corn cobs as a renewable substrate [26, 27], the titers and yields achieved in this study were about 2-fold less. However, the titers of 4-hydroxystyrene in this study were achieved with a simple glucose medium as a substrate in culture conditions using shake flasks. The results suggest that system used in this study as well as the achieved titers forms the basis for further improvement in the production of hydroxystyrene derivatives, and opens the possibility for a jar fermentation and extraction process. Furthermore, the previous report showed that the maximum concentration of 4-hydroxystyrene was limited to 3.3 mM because of the cytotoxicity of the product to the *E. coli* host [5, 28]. To alleviate the cytotoxicity of intermediates (phenolic acids) or products (hydroxystyrenes), a new host, such as solvent tolerant microbes, could be the solution for higher titers. For example, the solvent-tolerant *P.putida* S12 through a two-phase fermentation had higher than 10 g/L yields of 4-hydroxystyrene [8]. Further, if our approach described in this manuscript applies to the engineering of solvent tolerant microbes, such as *Pseudomonas* and yeast platforms, higher titer could be achieved [8, 9]. Furthermore, a fermentation platform using low-priced precursor sources, such agro-industrial wastes and lignocellulose materials [5, 27, 29], could be what is needed for the substantial and economical production of hydroxystyrene monomers, which are presently produced from non-renewable petroleum.

## Conclusions

The system in this study converts 4-coumaric acid to 4-hydroxystyrene through *pad* (phenolic acid decarboxylase), which is eventually converted caffeic acid and ferulic acid and then to 3,4-dihydroxystyrene and 4-hydorxy-3-methoxystyrene, respectively. *E. coli* strains expressing *tal* (tyrosine ammonia lyase) and *pad*, and *tal*, *sam5* (4-coumarate 3-hydroxylase), and *pad*, and *tal*, *sam5*, *com* (caffeic acid methyltransferase), and *pad* produced 4-hydroxystyrene, 3, 4-dihydroxystyrene and 4-hydroxy-3-methoxystyrene, respectively, using a simple sugar medium without precursor feeding. Furthermore, these pathways were extended in an *E. coli* strain with the biosynthesis machinery for overproducing tyrosine. Finally, the titers for 4-hydroxystyrene, 3,4-dihydroxystyrene and 4-hydroxy-3-methoxystyrene in the tyrosine-overproducing *E. coli* strains reached 355, 63, and 64 mg/L, respectively, in shaking flasks after 36 h of cultivation.

## Methods

### Bacterial strains, plasmids, and chemicals

The strains and plasmids used in this study are listed in Table 1. Antibiotics were added to the medium as required at the following concentrations: ampicillin, 100 mg/L; kanamycin, 50 mg/L; and tetracycline, 10 mg/L. Cinnamic acid, 4-coumaric acid, caffeic acid, ferulic acid, sinapic acid, 4-hydroxystyrene (4-vinylphenol), and 4-hydroxy-3-methoxystyrene (2-methoxy-4-vinylphenol) were purchased from Sigma–Aldrich (USA), and 3,4-dihydroxystyrene was purchased from Toronto Research Chemicals Inc. (Canada) as a standard for compound identification by HPLC.

**Table 1:** Plasmids and strains used in this study

Plasmid or strain	Relevant characteristics	Source
Plasmid		
pET-22b(+)	f1 ori, T7 promoter, Amp^R^	Novagen
pET-28a(+)	f1 ori, T7 promoter, Kan^R^	Novagen
pET-opTAL	pET-28a(+) carrying codon-optimized tyrosine ammonia lyase gene (*tal*)	Kang et al. [20]
pET-Sam5	pET-28a(+) carrying 4-coumarate 3-hydroxylase gene (*sam5*)	Choi et al. [17]
pET-COM	pET-28a(+) carrying caffeic acid methyltransferase gene (*com*)	Choi et al. [17]
pET-opT5	pET-28a(+) carrying *tal* and *sam5* gene	Kang et al. [20]
pET-opT5M	pET-28a(+) carrying *tal*, *sam5*, and *com* gene	Kang et al. [20]
pET28-tyrA*	pET-28a(+) carrying feedback-inhibition resistant chorismate mutase/prephenate dehydrogenase gene (t*yr*A^fbr^)	Kang et al. [20]
pET22-aroG*	pET-22b(+) carrying feedback-inhibition resistant DAHP synthase gene (*aro*G^fbr^)	Kang et al. [20]
pET-AG	pET-28a(+) carrying *tyr*A^fbr^ and *aro*G^fbr^ gene	This study
pET22-baPAD	pET-22b(+) carrying codon-optimized phenolic acid decarboxylase gene (*pad*)	This study
pET-opTD	pET-28a(+) carrying *tal* and *pad* gene	This study
pET-opT5D	pET-28a(+) carrying *tal*, *sam5* and *pad* gene	This study
pET-opT5MD	pET-28a(+) carrying *tal*, *sam5*, *com* and *pad* gene	This study
Strain		
*E. coli* DH5α	Cloning host	Invitrogen
*E. coli* C41(DE3)	Derivative strain of *E. coli* BL21(DE3)	Miroux and Walker [31]
*ΔtyrR*	*tryR* gene in-frame deletion mutant of *E. coli* C41(DE3)	Kang et al. [20]
ΔCOS1	*E. coli* C41(DE3); ΔtyrR::*tyrA* ^fbr^, *aroG* ^fbr^; tyrosine overproducing strain	This study

### DNA manipulation

The restriction enzymes (NEB; Takara), Ex Taq polymerase (Takara), pfu Taq polymerase (Enzynomics, Korea), an AccuPower Ligation kit (Bioneer, Korea), and a Quick & Easy *E. coli* gene deletion kit (Gene Bridges, German) were used according to the manufacturers’ instructions. The optimized tyrosine ammonia lyase gene (*tal*) and 4-coumarate 3-hydroxylase gene (*sam5*) from *S. espanaensis* and the caffeic acid methyltransferase gene (*com*) from *A. thaliana* were synthesized previously by DNA 2.0 [20]. Codon optimization and synthesis of the phenolic acid decarboxylase *g*ene (*pad*) from *B. amyloliquefaciens* DSM7 (GenBank FN597644) were performed with the GeneGPS™ program (DNA2.0).

### Construction of phenolic acid decarboxylase expression vectors and assembly of the artificial biosynthetic pathways

In order to construct an expression vector containing the phenolic acid decarboxylase gene (*pad*) that was under the control of independent T7 promoter, a 0.5-kb DNA fragment, which contained the synthetic *pad* coding region, was cloned into the *Nde*I and *Xho*I sites on pET-22b(+), which resulted in pET22-baPAD. The three genes (*tal*, *sam5*, and *com*) were independently cloned into pET-22b(+) or pET-28a(+) vectors [17, 20]. Using the *tal*, *sam5*, *com,* and *pad* genes as templates, four DNA fragments were amplified by PCR with the appropriate pairs of primers. In order to assemble the pET-opTD vector, the *tal* coding region was amplified using pET-opTAL as a template with the primer opTAL-F (5′-CATATGACCCAGGTGGTTGAACGCC-3′) and Cpac (the sequence is located downstream of the T7 terminator region of the pET vector and contains the designed *Pac*I site: TTAATTAATGCGCCGCTACAGGGCGCGTCC), also the *pad* coding region was amplified using pET22-baPAD as a template with the primer Npac (the sequence is located upstream of the T7 promoter region of the pET vector and contains the designed *Pac*I site: TTAATTAATCGCCGCGACAATTTGCGACGG) and baPAD-R (the sequence contains the designed *Xho*I site 5′-CTCGAGTTACTTCAGTTTACC-3′). Each of the amplified fragments were digested with corresponding sites and cloned between the *Nde*I and *Xho*I digested pET-28a(+) via ligation, which resulted in pET-opTD. A 2.5-kb *Pac*I fragment containing the *sam5* gene was PCR-amplified with the NPac and CPac primers using pET22-Sam5 as a template. The amplified fragment was digested with *Pac*I and cloned between the *Pac*I digested pET-opTD, which resulted in pET-opT5D. Finally, a 2.5-kb *Pac*I/*Spe*I fragment containing the *com* gene was PCR-amplified with the NPac and Cspe (the sequence is located downstream of the T7 terminator region of the pET vector and contains the designed *Spe*I site: ACTAGTTCCTCCTTTCAGCAAAAAACCCCTC) primers using pET22-COM as a template. A 2.5-kb *Pac*I fragment containing the *sam5* gene was PCR-amplified with the NPac and CPac primers using pET22- Sam5 as a template. Each of the two amplified fragments were digested with corresponding sites and cloned into *Pac*I digested pET-opTD, which resulted in pET-opT5MD (Figure 2).

### Construction of the l-tyrosine overproducing strain

An l-tyrosine over-producing strain of *E. coli* (ΔCOS1) was achieved by extra gene insertion of *aroG* and *tyrA,* feedback-inhibition resistance (fbr) genes on the *tyrR* gene locus. The genetic design of the *aroG* and *tyrA* feedback-inhibition resistance (fbr) genes was followed as previously described by *Lütke*-*Eversloh* and *Stephanopoulos* [25, 30] and used our previously made constructs [20]. The PCR product was generated using the *tyrA*^*fbr*^-*aroG*^*fbr*^-FRT-neo-FRT fragment as a template for pET-AG and FRT-neo-FRT fragment (Gene Bridges). We made a fragment containing both the *tyrA*^*fbr*^ and *aroG*^*fbr*^ gene cassette, in which the RBS and T7 promoter were positioned in front of each gene, through PCR with the following primers, IF-N1(5′-CGGTACCCGGGGATCACTAGTTGATCGGCGCGAGATTTAATCGCCGCGCAA T-3′) and IF-C1(5′-GTTAATTAAACTAGTCACGCTGCGCGTAACCACCACACCCGC CGCGCT-3′), and another fragment containing the FRT-neo-FRT using the following primers, IF-FRT1(5′-ACTAGTTTAATTAACCCTCACTAAAGGGCGGCCGCGAAGTTCCTATT-3′) and IF-FRT2(5′-CGACTCTAGAGGATCACTAGTAATACGACTCACTATAGGGCTCGAG GAAGTTCC-3′). These two fragments were connected between the *Spe*I site of pUC19 using the In-fusion kit (Clontech Laboratories, Inc., USA), resulting in pUC-AGFRT (Additional file 1: Figure S4). The 5.9-kb insertion PCR product was generated using pUC-AGFRT as a template and the following primers, tyrRr (5′-ATCAGGCATATTCGCGCTTACTCTT CGTTCTTCTTCTGACTCAGACCATATAATACGACTCACTATAGGGCTC-3′) and Inf-tryRfAG (5′-GTCATATCATCATATTAATTGTTCTTTTTTCAGGTGAAGGTTCCCATGC GTACTAGTCGTTCTACCATCGACACC-3′), and this gene cassette was inserted between the *tyrR* gene for gene insertional inactivation, which was done as previously reported using RED/ET recombination with a Quick & Easy *E. coli* Gene deletion kit (Gene Bridges). The clones growing on the kanamycin plate still contained the selection marker cassette, while all other clones containing insertional inactivation lost the selection marker. The kanamycin selection marker was removed from the chromosome by transforming the cells with an FLP recombinase expression plasmid, 707-FLPe (Gene Bridges). The insertional inactivation mutant (ΔCOS1) was verified through PCR using the following primers: tyrA-F (5′- CCATGGTTGCTGAATTGACCGCATTACG-3′) and aroG-R (5′- AAGCTTAACCACGA CGCGCTTTCACAGC-3′). The PCR product was sequenced and verified (Additional file 1: Figure S5).

### Culture conditions for production

Recombinant *E. coli* C41 (DE3) strains [31] harboring plasmids were grown at 37°C in a *Luria*–*Bertani* (LB) medium containing 50 μg/mL kanamycin. The overnight culture was inoculated (1.5%) into fresh LB medium supplemented with the same concentration of kanamycin. The culture was grown at 37°C to an optical density at 600 nm (OD_600_) of 0.6, and IPTG was added to the final concentration of 1 mM, and the culture was incubated for 6 h. The cells were harvested by centrifugation, suspended, and incubated at 26°C for 36 h in a modified synthetic medium (3 g/L KH_2_PO_4_, 7.3 g/L K_2_HPO_4_, 8.4 g/L MOPS, 2 g/L NH_4_Cl, 0.5 g/L NaCl, 0.1 ml/L Trace elements, 5 g/L (NH_4_)_2_SO_4_, 5 g/L MgSO_4_, and supplemented with 15 g/L glucose, 1 mM IPTG and appropriate antibiotics) [24]. For the feeding experiments, the cultures were supplemented with cinnamic acid, 4-coumaric acid, caffeic acid, ferulic acid, or sinapic acid (final concentration: 2 mM), respectively. The samples were collected after 36 h and analyzed by HPLC.

### Detection and quantification of the products

To quantify 4-hydroxystyrene, 3,4-dihydroxystyrene and 4-hydroxy-3-methoxystyrene, 1 mL of cell-free culture supernatants was filtered through a 0.2 μm cellulose membrane syringe filter (Sartorius) and used for HPLC analysis with a Dionex Separations module connected with a Photodiode Array detector (Dionex) set. Twenty microliters of the samples were applied to a J’sphere ODS-H80 column (4.6 × 150 mm i.d., 5 μm; YMC, Japan) in a high-performance liquid chromatography (HPLC) system [CH_3_CN-H_2_O (0.05% trifluoroacetic acid), 20–60% acetonitrile (CH_3_CN) for 25 min at flow rate of 1 mL/min; Dionex, USA] equipped with a photodiode array detector. Quantification of the three above-mentioned compounds was based on the peak areas of absorbance at 259 nm. Purchased 3,4-dihydroxystyrene and 4-hydroxy-3-methoxystyrene as a standard contains the impurity. Therefore, all the hydroxystyrene derivatives were measured as equivalent to 4-hydroxystyrene. For the quantification of 4-coumaric acid, caffeic acid, and ferulic acid, the HPLC analysis was followed as our previously described methods [20]. The data shown in this study were generated from triplicate independent experiments. The titers for each production were compared with single-factor ANOVA (P < 0.05) using the single-factor ANOVA tool.

## Additional files

**Additional file 1:** Further details of relevance to this study.
