# Supplementary material for: Artificial de novo biosynthesis of hydroxystyrene derivatives in a tyrosine overproducing Escherichia coli strain
Source: Microb Cell Fact. 2015 Jun 10;14:78. doi: 10.1186/s12934-015-0268-7 (PMC4460750; doi:10.1186/s12934-015-0268-7)
Supplement: Supplementary file 1 — Additional file 1: Further details of relevance to this study. [file 12934_2015_268_MOESM1_ESM.docx]

**Additional File 1: Further details of relevance to this study**

**Artificial *de novo* biosynthesis of hydroxystyrene derivatives in a tyrosine overproducing *Escherichia coli* strain**

**GC/MS identification of produced hydroxystyrene derivatives**

The recombinant *E. coli* cells harboring the pET22-baPAD plasmid produced the 14.5 min retention time peak with the 4-coumaric acid, which is the same retention time as the authentic 4-hydroxystyrene. Further GC/MS identification revealed that this compound had m/z 120.2, confirming the identity of the product as 4-hydroxystyrene. Additionally, a major peak was present at the same retention time (9.3 min) as the authentic 3,4-dihydroxystyene in the HPLC analysis and with a mass ion peak at m/z 136.2 using the caffeic acid. This peak confirmed the identity of the product as 3,4-dihydroxystyene. Finally, when using ferulic acid or sinapic acid as a substrate, it produced 15.2 min and 13.6 min retention time peaks, which were the same mass peaks at m/z 150.2 and 180.2 with 4-hydroxy-3-methoxystyrene and 4-hydroxy-3,5-dimethoxystyrene, respectively (Supporting Figure 1).

**Figure S1. Selected GC/MS data of bioconversion experiments**

(A) 4-hydroxystyrene (m/z 120.2) produced by 4-coumaric acid supplemented *E. coli* harboring the pET22-baPAD plasmid; (B) 3,4-dihydroxystyene (m/z 136.2) produced by caffeic acid, (C) 4-hydroxy-3-methoxystyrene (m/z 150.2) produced by ferulic acid, and (D) 4-hydroxy-3,5-dimethoxystyrene (m/z 180.2) produced by sinapic acid supplemented E. coli harboring the pET22-baPAD plasmid.


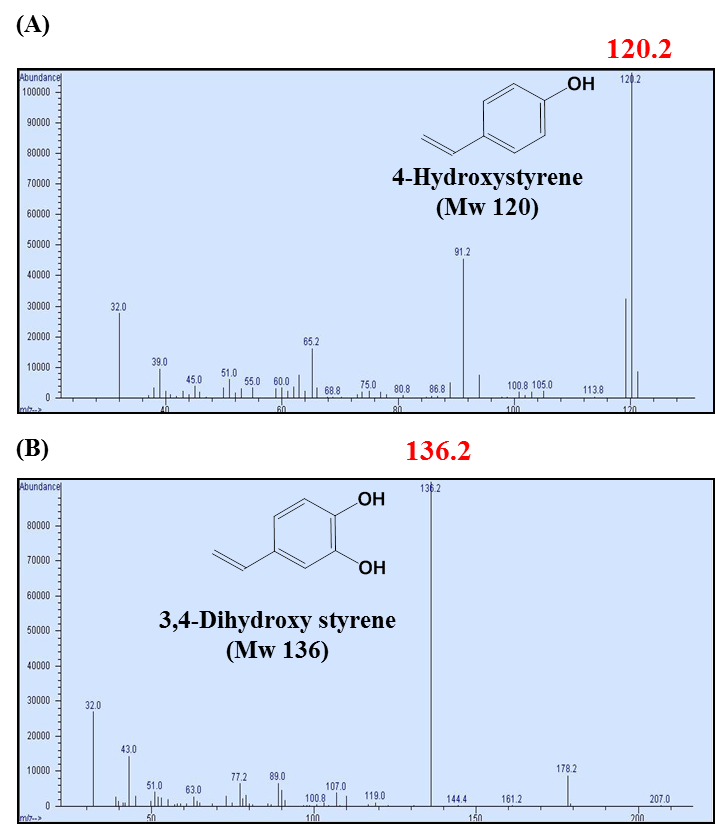


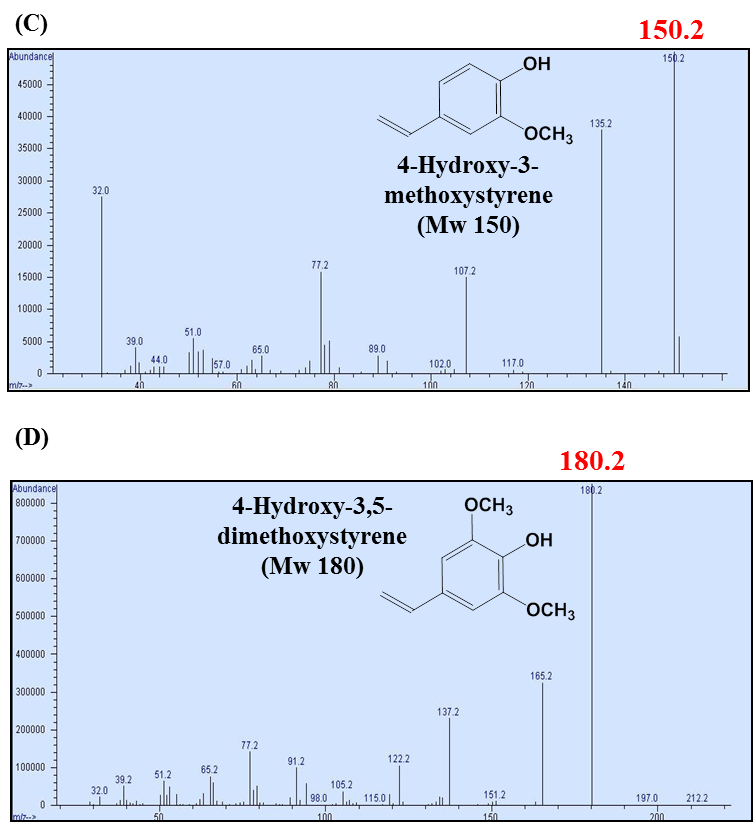


Figure S1

**
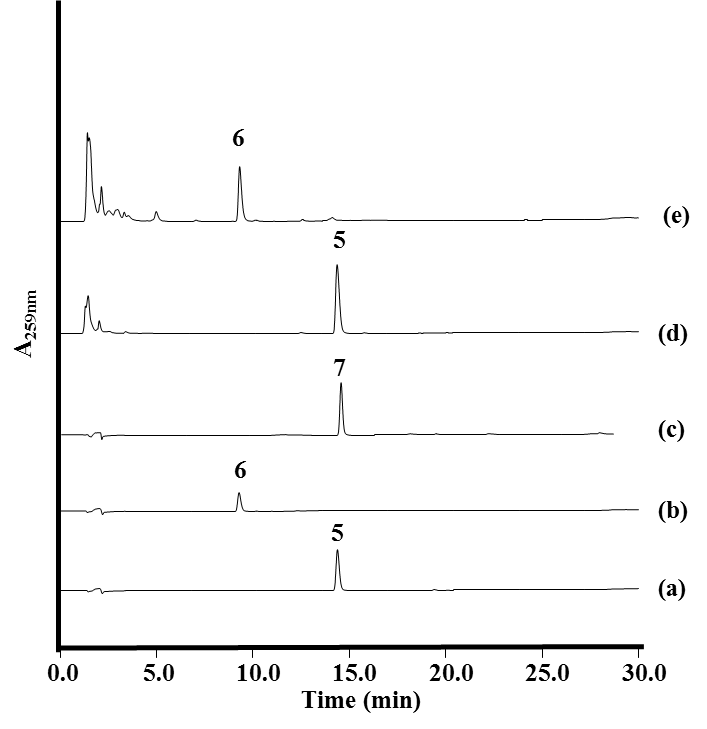
**

Figure S2.

**Figure S2. HPLC profiles of the production of 4-hydroxystyrene (d) and 3,4-dihydroxystyrene (e) by wild type *E. coli* strains expressed tal and pad (pET-opTD) and tal, sam5, and pad (pET-opT5D).** The absorbance was monitored at 259 nm. Peak 5, 4-hydroxystyrene; peak 6, 3,4-dihydroxystyrene; peak 7, 4-hydroxy-3-methoxystyrene. HPLC profiles of standard 4-hydroxystyrene (a); 3,4-dihydroxystyrene (b); 4-hydroxy-3-methoxystyrene (c); the culture broth of wild type E. coli harboring pET-opTD (d); pET-opT5D (e);


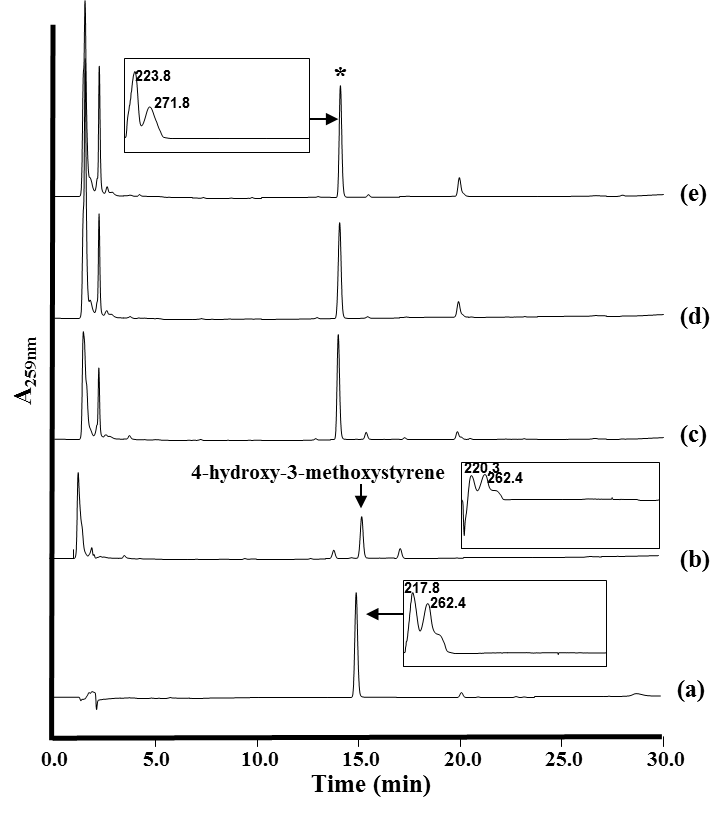


Figure S3

**Figure S3. HPLC profiles of the time-course cultivation (2, 12, 24, and 36 hours) of wild type *E. coli* harboring pET-opT5MD.** The cell was cultured in modified synthetic medium at 26°C for 2 hours (b), 12 hours (c), 24 hours (d), 36 hours (e). (a), profile of standard 4-hydroxy-3-methoxystyrene; *, unknown peak. The insets show the UV/Vis spectra of the compounds with the indicated HPLC peaks

**Confirmation of tyrR gene insertional mutant (ΔCOS1 ) by PCR**

The gene replacement of *tyrR*, a repressor gene of aromatic amino acid biosynthesis, with the *tyrA*^fbr^ and *aroG*^fbr^ gene cassette was constructed by RED/ET recombination (Supporting Fig 4). The insertional inactivation mutant (ΔCOS1) was verified with PCR using the following primers: tyrA-F (5’- CCATGGTTGCTGAATTGACCGCATTACG-3’) and aroG-R (5’- AAGCTTAACCACGACGCGCTTTCACAGC-3’). The PCR product was sequenced and verified. As result, a 3.1 kb of the PCR product with the tyrA-F and aroG-R primer set was detected from ΔCOS1 and the PCR product was not seen in the wild type. This result shows the insertion of the *tyrA*^fbr^ and *aroG*^fbr^ gene cassette in the *tyrR* gene (Supporting Fig 5).


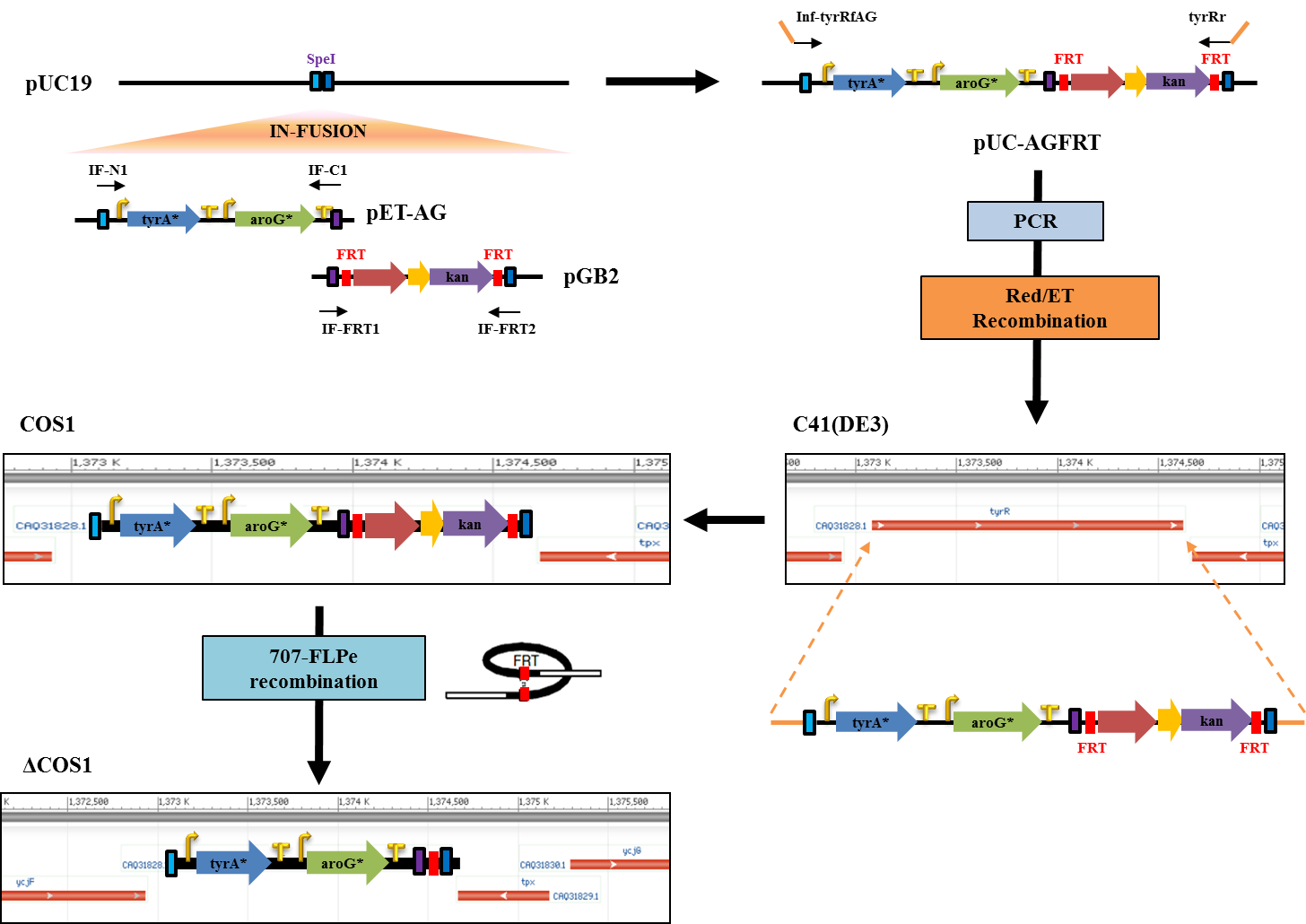


Figure S4.

**Figure S4. Strategy for the gene insertion in the *tyrR* region on the *E. coli* chromosome**.


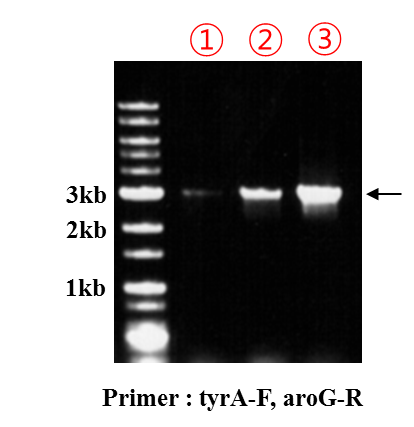


Figure S5.

**Figure S5. Confirmation of insertional gene inactivation by PCR**

**Analysis of L-tyrosine**

To quantify L-tyrosine, 1 mL of cell-free culture supernatants was filtered through a 0.2 μm Cellulose membrane syringe filter (Sartorius) and used for HPLC analysis with a Dionex Separations module connected with a Photodiode Array detector (Dionex) set. The L-tyrosine was separated on a YMC C18 column (150 × 4.6 mm, 4 μm). The following gradient was used at a flow rate of 1 mL/min: 5% to 80% acetonitrile for 25 min, 80% to 100% acetonitrile for 3 min, 100% acetonitrile for 3 min, 100% to 5% acetonitrile for 3 min, and 5% acetonitrile for an additional 3 min.


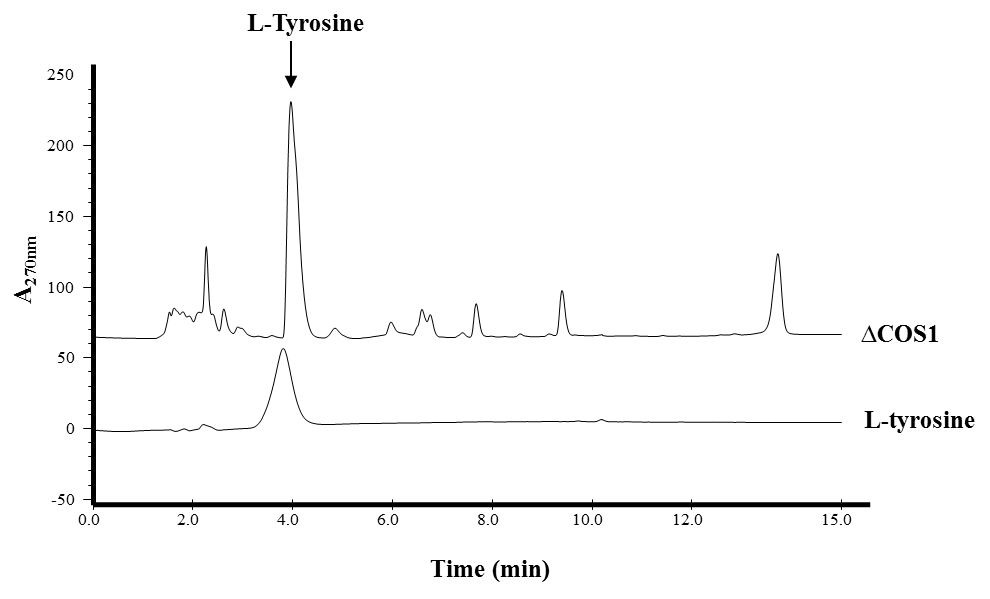


Figure S6.

**Figure S6. HPLC profile of L-tyrosine production in the engineered tyrosine overproducing strain (ΔCOS1)**

**
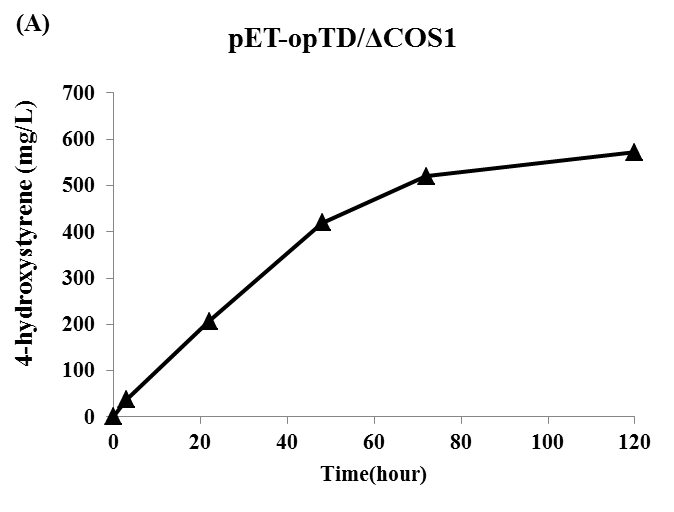
**

**
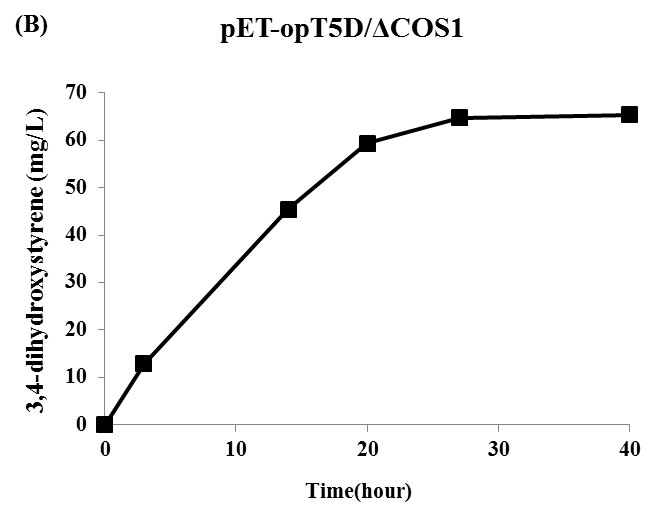
**

**
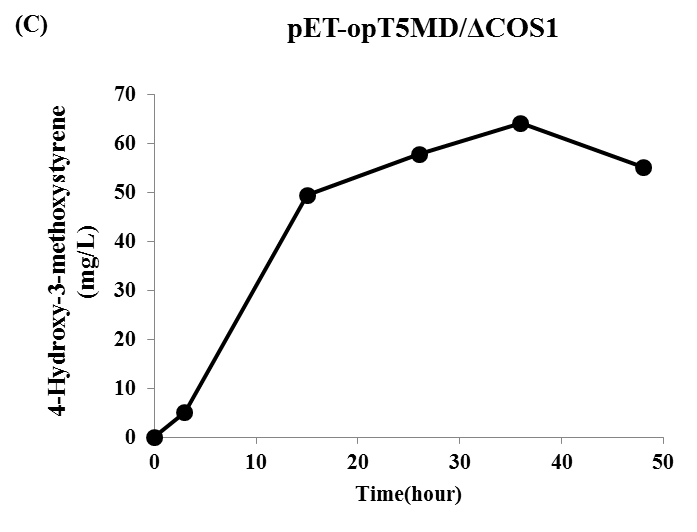
**

Figure S7.

**Figure S7. Accumulation of 4-hydroxystyrene, 3,4-dihydroxystyrene and 4-hydroxy-3-methoxystyrene in minimal media upon culturing of the tyrosine over-producing strains harboring pET-opTD (A); pET-opT5D (B); pET-opT5MD (C)**. The cells were cultured in modified synthetic medium at 26℃. ▲, 4-hydroxystyrene; ■, 3,4-dihydroxystyren; ●, 4-hydroxy-3-methoxystyrene. Compounds concentration quantified as absorbance area at 259 nm.
